# Organ-on-a-Chip: *Ubi sumus*? Fundamentals and Design Aspects

**DOI:** 10.3390/pharmaceutics16050615

**Published:** 2024-05-02

**Authors:** Ana Sofia Morais, Maria Mendes, Marta Agostinho Cordeiro, João J. Sousa, Alberto Canelas Pais, Silvia M. Mihăilă, Carla Vitorino

**Affiliations:** 1 Faculty of Pharmacy, University of Coimbra, 3000-548 Coimbra, Portugal; morais.asofia@gmail.com (A.S.M.); mariamendes1093@gmail.com (M.M.); martaisabel99@gmail.com (M.A.C.); jjsousa@ff.uc.pt (J.J.S.); 2 Coimbra Chemistry Centre, Department of Chemistry, University of Coimbra, 3004-535 Coimbra, Portugal; pais@qui.uc.pt; 3 Division of Pharmacology, Utrecht Institute for Pharmaceutical Sciences, Utrecht University, 3508 TB Utrecht, The Netherlands; s.mihaila@uu.nl

**Keywords:** organ-on-a-chip, microfluidic technology, design concepts, polydimethylsiloxane, stem cell

## Abstract

This review outlines the evolutionary journey from traditional two-dimensional (2D) cell culture to the revolutionary field of organ-on-a-chip technology. Organ-on-a-chip technology integrates microfluidic systems to mimic the complex physiological environments of human organs, surpassing the limitations of conventional 2D cultures. This evolution has opened new possibilities for understanding cell–cell interactions, cellular responses, drug screening, and disease modeling. However, the design and manufacture of microchips significantly influence their functionality, reliability, and applicability to different biomedical applications. Therefore, it is important to carefully consider design parameters, including the number of channels (single, double, or multi-channels), the channel shape, and the biological context. Simultaneously, the selection of appropriate materials compatible with the cells and fabrication methods optimize the chips’ capabilities for specific applications, mitigating some disadvantages associated with these systems. Furthermore, the success of organ-on-a-chip platforms greatly depends on the careful selection and utilization of cell resources. Advances in stem cell technology and tissue engineering have contributed to the availability of diverse cell sources, facilitating the development of more accurate and reliable organ-on-a-chip models. In conclusion, a holistic perspective of in vitro cellular modeling is provided, highlighting the integration of microfluidic technology and meticulous chip design, which play a pivotal role in replicating organ-specific microenvironments. At the same time, the sensible use of cell resources ensures the fidelity and applicability of these innovative platforms in several biomedical applications.

## 1. Introduction

The development of better models drives biological and biomedical research. These modern systems attempt to replicate human physiology and pathology across several levels, from the molecular level to the tissue and organ complexity. At this point, these systems generate a significant amount of information on the etiology of the disease, therapy strategies, and preventive measures. The human body, characterized by its intricate organization of cellular and non-cellular components, is a source of inspiration for developing diverse biological models. These models encompass many cell types and can mimic the organ systems to varying extents.

Conventionally, animal models are often used to recapitulate human physiology in several areas of biomedical research. However, the inherent differences between animals and human biology render these models unable to faithfully reproduce human responses due to numerous variables. While simplistic models, such as two-dimensional (2D) cell monocultures, offer usability in studying basic biology phenomena, they often fail to replicate the complex cell–cell and matrix–cell interactions that are critical for maintaining specific cellular phenotypes. In this way, 2D cultures cannot resemble tissues or organs. Three-dimensional (3D) cell aggregates, spheroids, and organoids cultures exhibit superior functional capabilities to 2D models, but they require further refinement to accurately simulate organ development and function [1,2,3,4]. Despite the ongoing advances, 3D models still have limitations in fully recapitulating cellular, tissue, and organ functions. Nevertheless, there is a pressing need to continue developing biological systems that can effectively address specific scientific questions in biomedical research [5].

The advances in microfabrication and microfluidics technology have revolutionized cell culture by providing a platform for generating complex and finely controlled microenvironments that closely mimic in vivo conditions [6]. Combining microfabrication and microfluidic technology makes it possible to precisely manipulate fluid dynamics, generating cellular microenvironments with relevant chemical gradients. These innovations have driven the emergence of alternative models known as organs-on-a-chip (OoaC), which integrate bioengineering, cellular biology, and microfluidics [7,8]. The OoaC systems enable the study of several biological and pathophysiological mechanisms of the human body in ways that were previously impossible with animal models and conventional 2D and 3D cell culture models [9].

**Figure 1:**
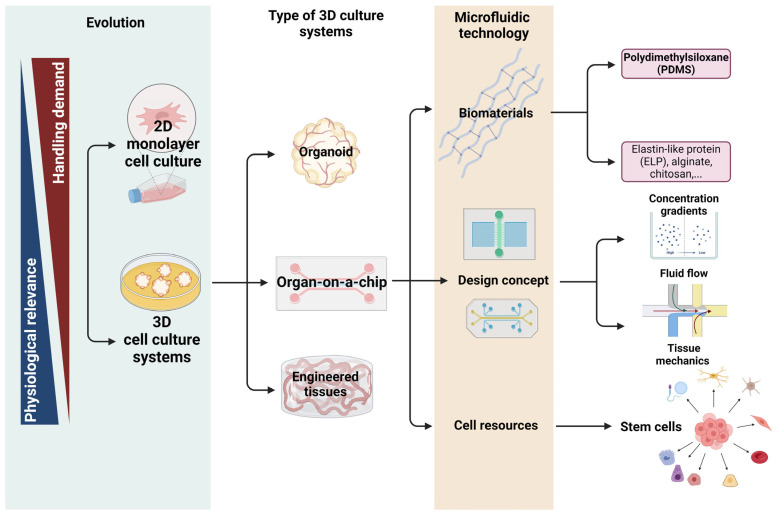
A perceptive flowchart highlighting the core themes explored in the review article, including the evolution of cell culture models, the exploration of some 3D culture systems, and the emphasis on organ-on-a-chip technology. Specifically, microfluidic technology was scrutinized, encompassing the biomaterials, design concepts (an integral aspect of organ-on-a-chip development), and cell resources, allowing for the consideration of diverse cell types and organ functionalities in manufacturing processes. Created from biorender.

This review will address the progress in cell culture models and the microfluidic culture system, exploring in more detail how they support the development of dynamic cell cultures with enhanced capabilities (see Figure 1). Additionally, it will discuss design considerations and describe the main components that are indispensable to recreating the cellular microenvironment using microfluidic chips.

## 2. Progression from Two-Dimensional Cell Culture to Organ-on-a-Chip

The conventional approach to pharmaceutical development presents some limitations concerning the screenings conducted to identify molecule(s) with real therapeutic benefit. The lengthy steps considered before the molecule is available for marketing, subject to approval by regulatory authorities, which, on average, last 10 to 12 years, constitute one of the critical challenges in developing a new drug and its translation to clinical practice [10,11]. This long period contributes to millions of deaths resulting from the quick progression of certain diseases, such as cancer. Furthermore, the emergence of new diseases driven by specific genetic mutations urgently requires fast and precise testing methodologies to evaluate the efficacy and safety of both novel and existing molecules, thus expediting the drug development process [10].

Recent years have shown a slowdown in the development of safe and effective drugs, primarily due to the failure to develop models that replicate the phenotype, function, and intercellular signaling of human cells in vitro. While animal models have traditionally been the benchmark in preclinical studies, their inadequacy in accurately mimicking the human drug metabolism hinders their predictive capacity for human outcomes [12]. Significant efforts have been made toward developing cell culture systems based on microengineering, microfabrication, bioprinting, and microfluidic technologies to address this challenge. These attempts aim to engineer models that are able to recreate controlled in vitro environments of 3D tissue systems, offering improved predictability and relevance to human physiology [13,14].

A comprehensive understanding of tissue development, function, and physiopathology needs consideration of how cells and tissues function within the context of intact living organs. Such organs comprise dynamic and heterogeneous tissues in terms of their 3D structure, biochemical microenvironment, and mechanical properties. Unfortunately, many studies on cell and tissue regulation rely on 2D cell culture models, which fail to reconstitute the complex cellular microenvironment found in vivo and cannot consequently demonstrate differentiated functions [14].

### Evolution of 2D Cell Culture and Addition of a 3D to the System

In 2D cultures, the cells are grown as a monolayer on a flat surface, a method that has been employed for many years and remains a valuable tool for drug screening, toxicity assessments, and the study of the cellular and molecular mechanisms of numerous diseases, such as Parkinson’s Disease, diabetes, and cancer, among others [15,16,17]. The paradox of 2D cell cultures arises from their simplicity, stability, ease of handling, and high performance (see Table 1). Furthermore, gene editing technology has facilitated the development of induced pluripotent stem cell lines (iPSC) from a single donor, enabling the derivation of multiple tissue types with a consistent genetic background [15].

However, despite widespread use, 2D cell culture models have inherent limitations, preventing them from accurately mimicking human physiology. Plate-cultured cells exhibit inconsistent properties compared to their original tissue, leading to the loss of standard tissue architecture, the absence of biophysical forces, and the 3D heterotypic environment or niche found in vivo. These differences are crucial, as cells in their natural environment dynamically interact with other cell types within the highly organized structure of the extracellular matrix (ECM) [18]. Such unnatural conditions can significantly alter the phenotypic properties of cells, limiting the precise similarity of physiological manifestations in vitro. These differences become apparent when comparing in vitro drug diffusion kinetics to in vivo conditions, where effective doses in 2D cultures often prove ineffective when scaled for animals and patients.

**Figure 2:**
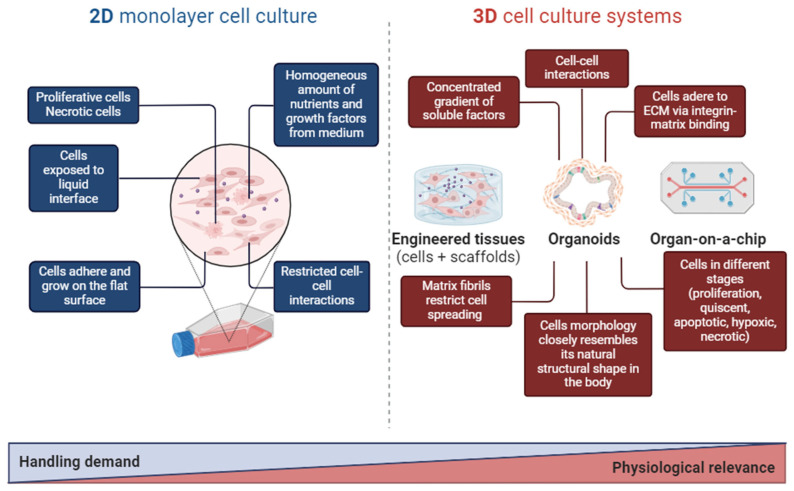
Main differences between 2D and 3D tissue platforms.

Efforts to address these limitations have led to the development of 3D cell culture models, where cells are embedded within ECM gels [14]. This approach enhances the expression of differentiated functions and promotes tissue organization. However, 3D culture models still fall short of fully replicating the characteristics of organs in vivo, including critical features such as the tissue–tissue interface (vascular epithelium–endothelium), spatio-temporal gradients of chemicals and oxygen, and a mechanically active microenvironment (movements of inhalation and exhalation—respiration). Additionally, 3D culture systems face technical challenges, such as evaluating physiological diffusion gradients (transport of ions in the kidney) or collecting samples of secreted cellular products (bile flow in the liver). Given the limitations of both 2D and 3D cell culture models, the analysis of normal and pathological cellular processes often relies on studies using animal models despite this being expensive and time-consuming [19]. Nevertheless, it is crucial to acknowledge the advantages and disadvantages of both 2D and 3D cell cultures and tailor their use according to the specific application (Figure 2).

**Table 1:** Comparison between 2D and 3D tissue platforms in terms of biological complexity, manufacturing, and outputs (adapted from [13,20]).

	2D Monolayer Cell Culture	3D Cell Culture Systems		
		Engineered Tissues (Cells + Scaffolds)	Organoid	Organ-on-a-Chip
**Proliferation**	Often proliferate at a faster rate than in vivo	May proliferate at a faster/slower rate compared to 2D cultured cells depending on cell type and/or type of 3D model system		
**Stage of the cell cycle**	Cells are likely to be in the same stage of the cell cycle	3D model contains proliferating, quiescent, hypoxic, and necrotic cells		
**Gene/protein expression**	Cells display differential gene and protein expression levels compared to in vivo models	3D model exhibits gene/protein expression profiles more similar to those of **in vivo** tissue		
**Production method**	Differentiated, grown on rigid flat surfaces as a monolayer.	Fabricated with scaffold and casting mold.	Embedded in matrigel to self-organize.	Seeded in engineered chambers with perfusion.
**Maturation**	Immature	Improved; still lacking	Improved; still lacking	Improved; still lacking
**Cell morphology and type**	Usually monotype, not resembling physiological conditions	Size and shape similar to in vivo	Size and shape similar to in vivo	Depends on platform design
**Extracellular matrix (ECM)**	Limited composition	Similar to in vivo	Similar to in vivo	Depends on platform design
**Tissue architecture**	Absent	Simple	Complex, similar to organs’ developmental stages	Complexity depends on platform design
**Diffusion of signal factors and nutrients**	Short distances (through cell membranes)	Concentration gradients may exist (may be affected by ECM properties)	Inner cells may die or lack maturity due to ineffective transport to the interior	Precisely controlled temporal and spatial gradients
	Unlimited access to oxygen, nutrients, metabolites, and signaling molecules	Variable access to oxygen, nutrients, metabolites, and signaling molecules		
**Perfusion**	No	No	No	Yes
**Variability**	Low	High	High	High
**Reproducibility**	High	Low	Low	Sometimes low
**Controllability**	High	Low	Very low	Very high
**Use**	Easy	Difficult	Easy	Difficult
**Characterization and analyses**	Limited, easy retrieval, except for cells	Tissue function analyses are possible, but cell retrieval and phenotypic analysis can be hard	Tissue function analyses are possible, but cell retrieval and phenotypic analysis can be hard	Real-time tissue/organ function analyses are possible with easy cell retrieval

## 3. Organoids

Organoids refer to 3D cell cultures with multiple cell layers, typically derived from stem cells obtained from biopsies or pluripotent stem cells. These cell cultures can self-organize and form tissue or organ-as structures, although in a simplified model. Organoids are complex and provide a singular opportunity to recreate human organ anatomical structures at the microscale while mimicking basic functions or simulating diseases closely resembling in vivo conditions. The applications of organoids in both basic and translational research are extensive, encompassing fields such as biomedical research, drug discovery, biobanking, gene profiling, and regenerative medicine. Organoids demonstrate remarkable in vitro–in vivo correlations, providing their effectiveness in testing drug efficacy and toxicity in disease models (e.g., cancer, genetic disease, infection, or immunomodulation). Additionally, organoids enable the evaluation of the cell resistance mechanism of several drugs, which is useful for the development of a personalized medicine strategy and the establishment of living biobanks [21,22,23,24,25,26,27,28]. Thus, organoids provide a robust platform for studying numerous diseases, effectively bridging the gap between animal models and humans [29]. Various organoids are created through different types of cell culture systems. These include 3D-bioprinted scaffolds, organ-on-a-chip, and microfluidics-based 3D cell culture models. The next section will provide detailed insights into organ-on-a-chip and microfluidics-based 3D models.

## 4. Organ-on-a-Chip

Diverse cell types and ECM components are organized within pre-production chambers equipped with microfluidic channels, enabling the controlled flow of fluid through the cells. These cells are cultivated under different conditions, facilitating the simulation of tissue complexity. This process corresponds to developing microphysiological systems (MPS)—such as OoaC. These models offer precise control over tissue architecture and composition, allowing for the pre-programming of specific oxygenation and nutrient diffusion profiles. These achievements have substantially enhanced the viability of the chip, enabling its sustained functionality for several weeks to months [30]. Table 2 presents the advantages and drawbacks of organs-on-a-chip (OoaC). The main advantage of these systems is the ability to test new medicines at a small scale in human tissues, preserving their intrinsic tissue variability. This promises to accelerate research processes while diminishing the reliance on in vivo models and associated resources. MPS promote cellular differentiation and maturation, and acquisition data concerning tissue function. These systems facilitate cell–cell interactions, validate specific tissue interactions, and expose cells to different biophysical forces (shear stress, mechanical tension, electrical forces, peristalsis, and respiratory movements). As a result, MPS offer an increase in biological reality compared to the 2D culture systems discussed earlier.

Another advantage of MPS is their ability to integrate multiple biosensors. These biosensors facilitate a wide range of functions, including genetic sequencing, monitoring analytical parameters such as pH, oxygen levels, glucose, lactate, and temperature, assessing cell metabolism (including cytokines, metabolites, and secreted biomarkers), gauging organ activity, assessing barrier integrity, and measuring metabolic parameters [31,32,33,34,35,36,37,38]. Moreover, MPS can respond to external stimuli, which can be electrical, optical, or mechanical, and offer real-time monitoring and sample recovery capabilities. This rich array of biosensors provides essential information for maintaining a precise and reproducible cell culture environment characterized by high specificity and sensitivity. In turn, an important drawback to be considered is the impact of the surface effect on the performance of these systems. Due to the small dimensions of the fluids involved, surface effects exert a predominant influence over volume effects. These can compromise the accuracy and precision of the analysis, with specific desired molecules being susceptible to adsorption. Additionally, the laminar flow at the convergence of multiple fluids further complicates the mixing process, potentially leading to incomplete or inadequate homogenization.

### 4.1. Fundamentals

To develop reliable cell cultures established on microfluidics, it is necessary to replicate the cell microenvironment observed in vivo. Achieving an in vitro biomimetic cell microenvironment requires integrating microfabricated substrates with microfluidic technology and cell biology in complex, multifunctional designs. Cells and the ECM produce different biochemical and mechanical signals analogous to the in vivo microenvironment. These signals are crucial for the structuration of the tissue organization and growth, establishing cell polarization and migration, maintaining a balance between cell proliferation and apoptosis, and regulating cell behavior and protein expression [7]. Cell–cell communications within the cellular microenvironment are characterized by a reduced communication distance, continuous nutrient supply, waste removal, and synergistic responses to external stimuli. Microfluidic systems emerge as a valuable tool for reconstructing the cellular microenvironment, enabling the precise control of intercellular communication and the application of biochemical and biophysical stimuli essential for tissue or organ formation and maturation [39,40].

**Figure 3:**
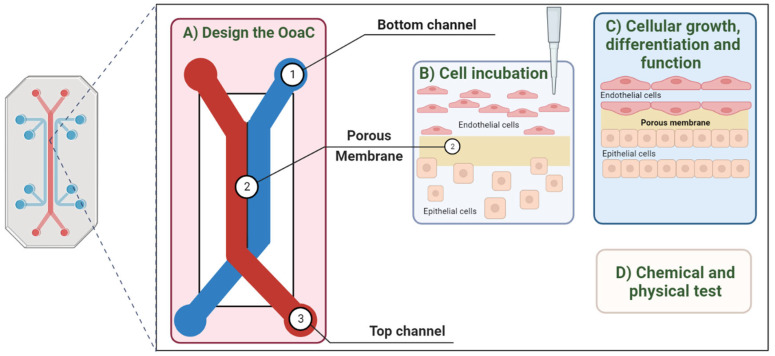
Key steps involved in the manufacturing process of an OoaC. The method of producing different OoaCs is the same, considering its application. (**A**) The design of the platform must be in line with the properties that the system intends to address. (**B**) Different cells must be incubated in the device. (**C**) Cell growth, differentiation, and function are established so that the chip functions as an organ. (**D**) The data are obtained through tests that allow for changes to be detected in the system. [Adapted from [44]].

OoaC represents a 3D cell culture model integrating microfluidic technology with tissue engineering. In this system, cells are meticulously arranged and cultivated within chambers and channels continuously perfused to mimic the physiology of tissues and biological organs in realistic models (Figure 3). OoaC technology shows the capacity to replicate the functionalities of an organ, including the complex multicellular architecture, human physiology, critical aspects such as cell–cell and matrix–cell interactions, physicochemical microenvironment, the vascular system, and electrical stimulation, features impossible to replicate in 2D and 3D in vitro culture static systems [41]. This micro-scale system finds applications in disease modeling, drug screening, electrophysiology, and identifying patient subgroups that may benefit more from a given clinical treatment. Moreover, OoaC technology supports the real-time monitoring of cells and tissue-specific responses in a non-invasive manner, which require continuous monitoring to assess tissue functionality and responses to environmental stimuli, including pathogens, drugs, or toxic compounds [42,43].

Initially, OoaC incorporated different analysis techniques, such as the enzyme immunoabsorption assay (ELISA), electrical transepithelial resistance (TEER), and multiple electrode matrices (MEA) [45,46,47]. Currently, distinct techniques are applied concomitantly on a single OoaC platform, including physical (temperature, pH detection), chemical (immunobiosensors to monitor secreted biomarkers or to assess oxygen levels), and optical (sensors to observe the neuromuscular contractile capacity). These sensors allow for the continuous monitoring of the environment and cellular behavior. However, ensuring the long-term stability of the embedded sensors requires technical improvements to prevent loss of accuracy or precision during prolonged cell culture times. Progress in this technology reveals the growing use of MPS in various contexts, offering detailed information on cellular functions not achievable with other platforms [48].

### 4.2. Key Components

The development of Ooac platforms relies on four major components: microfluidics (the fluid that provides all the components necessary for cell maintenance and balance, and which allows the removal of the residual liquid with surplus cells); the living cell component (2D or 3D cell structure often associated with biomaterials such as hydrogels); the physicochemical stimulation (application of stimuli to mimic in vivo microenvironment); and the detector (sensors and analysis technologies for the collecting and processing of relevant data). These components are essential for creating functional and coordinated tissues within microphysiological devices.

### 4.3. Organ-on-a-Chip vs. Organoid: Synergistic Potential

OoaC development involves specific design and production characteristics, whereas organoid formation relies on the stochastic principles of self-organization. Organoids formed from induced pluripotent stem cells (iPSC) or human biopsies mimic the architecture and functioning of in vivo tissues [49]. However, these improvements exhibit challenges such as long development times, poor reproducibility, and limited monitoring capabilities. For example, the iPSCs are differentiated in vitro to respond to specific inductive stimuli. However, they acquire a 3D structural arrangement that contains different cell types in spatial configurations like those recorded in organs during normal in vivo development [50]. Organoids derived from human biopsies are also associated with some limitations, including inadequate oxygenation and a reduced blood flow, which result in escalating necrosis within the core [50]. This condition results in decreased viability and a shortened lifespan for the organoids. They also fail to represent high-fidelity cell types accurately, have poor reproducibility, limited maturation and an atypical physiology, and lack spatial organization [51]. The complexity of the sample collection process, compounded by the challenge of reading and analyzing relevant tissue development parameters and the difficulty of assessing these cultures, makes real-time monitoring difficult. These features may restrict their reliability in certain applications. A critical evaluation of tissue-on-a-chip technology and organoids is summarized in Table 1.

To merge the best of both approaches, a novel 3D in vitro model was constructed—synergic engineering [52]. The design of this new model shows the precision of bioengineering, typically produced in an organ-on-a-chip, and incorporates the spontaneous formation and organization at random of the common development of organoids. This combination is promising for the development of a culture medium capable of addressing the diverse needs of connected tissues, similar to blood in the multi-organs system [53,54,55].

### 4.4. Microfluidic Technology

The progress of microfluidic technology has attracted attention from researchers across diverse fields of knowledge, owing to its capacity to construct systems with tailored structures for evaluating the physical relevance of fluid mechanisms and the transport of fluids. There have been significant advances in the manufacturing, the surface materials, and the integration of different operations and techniques [19].

Microfluidics encompasses the precise manipulation and processing of fluids at a micro-scale using a network of predefined channels within a device, which can range in size up to hundreds of micrometers. Its application involves integrating it into a device to recreate the physiological microenvironment of tissues and/or organs in vivo, whether in normal or diseased conditions. This capability derives from its control over fluid and its spatial–temporal physical characteristics [56]. Initially used for biochemical analysis and detection, microfluidic or lab-on-chip systems have more recently been found to be applicable in diagnosis. These devices facilitate the study of some parameters, conditions, and biological mechanisms in vitro, while consuming minimal quantities of reagents and cells, yielding highly sensitive results in a short period of time, distinguishing them from conventional cell cultures [57]. Microfluidic systems complement in vivo strategies and demonstrate their value in drug development and biological research, offering opportunities for evaluating and developing novel biocompatible materials [58].

In microfluidics, fluids are directed to flow through a microchannel by applying pressure to a connected reservoir. Flow within microfluidic devices is characterized by a low Reynolds number, indicating laminar flow where inertia forces are negligible compared to viscosity forces [59].

#### 4.4.1. Manufacturing Components

Microfluidic culture devices are commonly fabricated using the soft lithography technique, employing polydimethylsiloxane (PDMS) [60,61]. This method begins with a mold commonly created from photoresistant silicon containing the negative image of the desired channel pattern. The mold is then filled with the material to form the chip shape and a cross-linking agent. Depending on the intended use, the resultant block can be sealed reversibly or irreversibly following cooling and separation. Soft lithography facilitates the production of multilayer microfluidic devices with flexible microstructure configurations, representing a rapid prototyping technique for constructing micro- and nanostructures with a defined channel pattern, mostly by applying a low-cost polymer. This process represents an evolution of previously employed methods, incorporating microelectromechanical system techniques [43,62].

However, the time and cost associated with the soft lithography method make it unsuitable for mass production. The chip development process involves multiple steps with several constraints, and fully automating certain process phases (e.g., assembly and gluing) is challenging. Consequently, devices produced through this technique exhibit a much lower structural complexity than in vivo tissues. As an alternative, 3D printing has emerged as a promising technology for microfluidic devices [63,64,65]. With 3D printing, complex and realistic structures can be rapidly produced at a low cost. This method allows for the creation of microfluidic systems with the desired configuration in a single step using only one device, where digital data are converted into the desired structure. Moreover, 3D printing enables procedural standardization and yields more reproducible devices compared to traditional method [66].

Furthermore, 3D printing offers the possibility of bioprinting microfluidic models in vitro, allowing for the direct bioprinting of 3D artificial tissues onto microfluidic platforms [67]. This capability facilitates the production of tissues with intricate microstructures, enabling a thorough investigation of various stimuli, including dynamic mechanical cues (rigidity and fluid flow) and chemical cues (chemotaxis and concentration gradients) [65].

#### 4.4.2. Biomaterials—Application in Microfluidic Systems

The remarkable advancement of biomaterials facilitates their use in in vitro microenvironments, permitting the simulation of cell niches in vivo. Moreover, they prove invaluable in tissue engineering for regenerating and replicating the various types of normal and diseased tissues in tissue engineering. The increasing development of 3D culture models applying biomaterials has demonstrated significance in the creation of biological models for the study of complex biological processes, which are beyond the scope of traditional 2D models [68].

PDMS, known as a synthetic polymer with high optical transparency, facilitates the observation of cell behavior and the detection of cell molecule expression responsible for various pathophysiological mechanisms. Incorporated via a brightfield or fluorescence microscope, PDMS enables efficient cellular manipulation by integrating microvalves within the chip. Its notable biocompatibility with the cellular material ensures the long-term viability of an in vitro cell culture within microchannels or microchambers. Furthermore, its gas permeability is deemed advantageous for cell development [69,70]. However, PDMS hydrophobic characteristics hinder cell adhesion in these systems. Alternatively, other biomaterials, including collagen, fibrin, agarose, and synthetic polymers—polyethylene glycol (PEG)—offer configurable microstructures for the development of microfluidic cell cultures [71]. These materials display promising abilities to mimic physiological conditions and adopt the development of cell structures similar to those observed in the ECM in vivo [72,73,74,75].

#### 4.4.3. Application of Dynamic Systems in 3D Culture Models

Initially, microfluidic systems applied to cell culture involved a single cell layer aligned with the microchannels or microchambers with controlled medium perfusion. The progression of these systems permitted the investigation of cell growth, proliferation, differentiation, and drug screening. Gradual modifications to these analytical structures facilitated the development of in vitro perfused 3D cell cultures, representing a significant step towards mimicking in vivo dynamics, as flow influences the structural complexity of in vitro cells [76,77]. Microfluidic 3D cell cultures have several advantages, including the regulation of cell size, tissue reproduction, and the ease of manipulation of different hydrogels [78]. Moreover, these platforms demonstrate considerable potential for efficient high-performance experimentation [79]. Given the increasing demand for natural products, which are significant sources of new chemical entities (NCE), microfluidic systems can play a crucial role in understanding and assessing the impact of these compounds. By identifying the physiological action of specific compounds within original complex matrices, microfluidic systems aid in identifying potential drug candidates [80].

As evidenced, microfluidic devices provide novel models for diverse areas, such as studying biological disease mechanisms, identifying new targets, screening new drugs, and developing new biomaterials. The continuous advances and efforts to create models that better replicate the in vivo situation have led to the emergence of the technology now known as organ-on-a-chip [81,82].

### 4.5. Design Concepts

**Figure 4:**
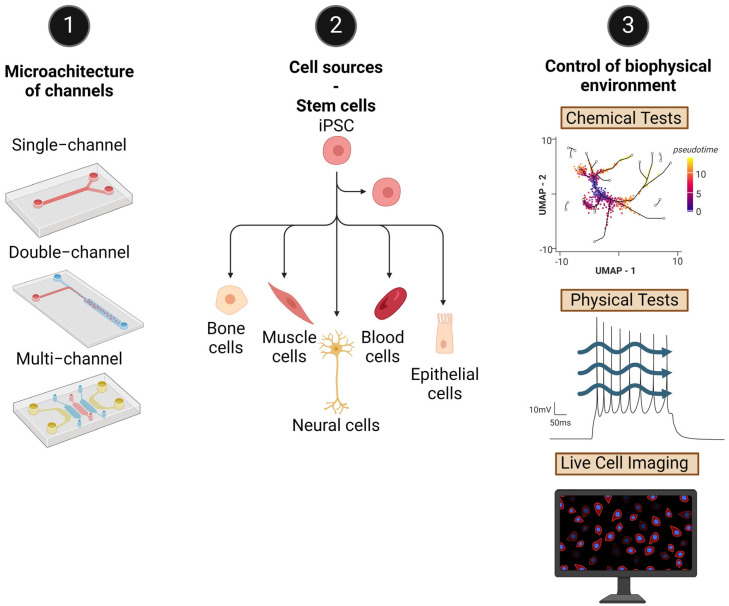
Design of different parameters for OoaC systems to mimic specific features of tissue microenvironments. 1. The microarchitecture of the channels is the most important feature to consider. This design represents biomechanical behavior and interactions between cells that are similar to those of the tissue of interest. 2. The cell sources are also relevant because they should closely mimic the cellular composition and behavior of human tissues or organs. This ensures that the OoaC models accurately represent the physiological responses and functions of the human body. In the case of disease modeling or drug screening, selecting cell sources derived from patients can provide valuable insights into disease mechanisms and drug responses. Disease-relevant cell sources enable the development of personalized OoaC models tailored to specific patient populations. 3. Control of the biophysical environment. The assessment and control of biomechanical, electrical, and biochemical properties in OoaC models should be carefully conducted because of the influence of cell behavior, tissue organization, and physiological responses. Biomechanical properties such as stiffness, elasticity, and substrate topography play a significant role in cell–substrate interactions, cell adhesion, migration, proliferation, and differentiation. Understanding fluid dynamics is essential for the design of OoaC systems with physiologically relevant flow conditions and optimizing of culture conditions for cell viability and functionality. Several tissues and organs, such as the heart and nervous system, are electrically active. Therefore, incorporating electrical stimulation into OoaC systems can enhance their physiological relevance and enable the study of electroactive tissues and organs. Biochemical properties, including signaling molecules, growth factors, and cytokines, play a critical role in cell–cell communication, tissue development, and physiological responses. Comprehending their biochemical properties helps to optimize culture media composition, incorporate relevant signaling cues, and study cell signaling pathways within OoaC models.

The development of OoaC involves the careful consideration of several critical parameters, including the geometry (whether a single channel, double channel—parallel or sandwich configuration—or multiple channels), dimensions (ranging from millimeters to submicrons), channel shapes (circular or rectangular), and the biological context (encompassing cell microenvironments, cell–cell interactions, and biomechanical and biophysical factors); see Figure 4. It is essential to strike a balance between increasing biological complexity, which enhances physiological relevance, and the experimental challenges associated with this. One significant advantage of meticulously controlling these characteristics is the ability to design OoaC systems that effectively monitor flow properties and biomechanical factors. The combined impact of these considerations significantly enhances in vitro testing and positions this tool as a preferred approach to 2D cultures or simpler 3D models. The following section will detail the biological context of OoaC.

#### 4.5.1. Cell—Extracellular Matrix Interaction

The process of tissue formation is facilitated by ECM proteins secreted by various cell types, providing essential physical support to the structure. These proteins promote interactions between cells and matrix, guide cell development and behavior, and activate intracellular signaling pathways. MPS can incorporate the ECM, facilitating the creation of gradients of several parameters to accurately simulate the in vivo environment. Some studies have illustrated the different environmental factors, such as oxygen gradients, nutrients, and soluble factors. For example, developed microfluidic devices have studied the effects of collagen on the growth and differentiation of mesenchymal stem cells and studied the impact of polyornithine and laminin on the proliferation and phenotypic differentiation of rat neural progenitor cells [83,84,85,86].

As an illustration of this, a microfluidic device was designed to simulate the microenvironment of neuronal growth by incorporating gradients of soluble guidance cues alongside surface-bound guidance signals [87]. The laminin gradient on the surface allowed for the modulation of the neuronal growth cone in polarity in response to the gradients of neurotrophic factors. This model provided valuable insights into the mechanisms underlying tissue formation and progression, highlighting the importance of understanding these processes for advancing biomedical research [87,88].

#### 4.5.2. Cell–Cell Interactions

Cell–cell interactions play an important role in morphogenesis, cell development, and tissue healing, as evidenced by their influence on the intricate organization of the human body. Achieving the complex, ordered, and synergistic arrangement of various cell types is essential for forming organized structures with functional interactions. These interactions can occur through direct contact—the cell–cell junction—or indirect contact—the diffusion of soluble factors—under normal physiological conditions. Some OoaC devices have been explicitly constructed to investigate the impact of interactions between different cell types. This technology enables the manipulation and cultivation of diverse cell types within a microchamber, highlighting the significance of cellular communication in regulating functions and the migration of metastatic cells [43]. Such studies emphasize the critical role of cell–cell interactions in maintaining tissue homeostasis and understanding disease processes.

#### 4.5.3. Control of the Biochemical Environment

##### Concentration Gradients

Some cellular behaviors, including growth, differentiation, migration, and angiogenesis, are not only influenced by cellular interactions but also by biochemical factors present in the tissue microenvironment. These factors exert crucial regulatory actions and form gradients of soluble concentrations [89,90]. While these gradients are physiologically relevant, they are challenging to simulate in 2D and 3D cell cultures. OoaC offers a solution to this limitation by generating chemical gradients that mimic those found in vivo, facilitating the study of their impact on cell behavior orientation and evaluating their significance in the variability of biological responses. Microfluidic cell culture systems have been specifically developed to investigate angiogenesis in the presence of growth factor gradients and to validate the chemo-attraction of leukocytes exposed to distinct concentrations of inflammatory stimuli [91].

Furthermore, oxygen gradients have been identified as crucial factors in tissue performance and homeostasis maintenance, promoting angiogenesis and inducing an acute cellular response in inflammatory situations [92]. These findings highlight the importance of understanding and accurately replicating microenvironmental gradients in cell culture models for comprehensive biomedical research and drug development.

#### 4.5.4. Control of the Biophysical Environment

##### Fluid Flow-Induced Stress

Fluid flow serves the crucial function of mass transport, facilitating the administration of nutrients, the distribution of soluble factors, and the collection of cellular waste throughout the human body via blood vessels and lymphatic vessels. The fluid flow velocity varies depending on the organ and its location within the body, potentially inducing diverse responses in different cell types. OoaC systems enable the generation and simulation of fluid shear stress (FSS) in microchannels, which activate cellular surface molecules and the associated signaling cascades. This allows for the evaluation of the FSS effect on cellular adhesion, growth, morphology, and protein expression [93]. Simulating these stresses in models representative of human physiology is of high significance, enhancing the investigation of their regulatory effects on specific tissues. Several studies have revealed the impact of the FSS on modulating cell behavior, including cytoskeleton reorganization, and its role in shaping angiogenesis associated with tumor biology [94]. By mimicking physiological fluid flow conditions, OoaC systems offer valuable insights into the complex interplay between mechanical forces and cellular responses, advancing the understanding of tissue physiology and disease mechanisms.

##### Tissue Mechanics

Under both physiological and pathological conditions, cells experience organ-specific mechanical stimuli such as traction and compression forces, in addition to the FSS. To simulate and interpret these forces, a multilayer microfluidic device was developed to simultaneously assess the effects of solid mechanical and surface tension stress induced by cyclic wall elongation, replicating the air–liquid interface in lung alveoli [95,96]. Similarly, in intestinal absorption and metabolism studies, a microfluidic chip was designed in which intestinal epithelial cells faced physiological mechanical deformation, including drip flow and cyclic mechanical distortion. These mechanical stimuli prompted the spontaneous formation of robust intestinal villi structures and facilitated the differentiation of these cells into various cell lines found in the small intestine. Mechanical stimulation emerges as a crucial element of differentiation during the physiological process [97,98,99].

Overall, these systems hold huge potential for advancing studies related to tissue engineering by mimicking organ physiology and analyzing both healthy and diseased tissues, as well as their underlying etiology. This technology has demonstrated significant promise in investigating specific molecular mechanisms, facilitating efficient drug screenings, assessing toxicity, and contributing to predictive medicine. These aspects are considered pivotal in the contemporary drug development process [10].

## 5. Cell Resources for Developing an Organ-on-a-Chip

Cell models should accurately replicate the functions of the tissue or organ they represent and maintain the correct ratio between the different cell types comprising the structure. A careful selection of biological resources is essential to achieve a high degree of analogy between OoaC and human tissues. Immortalized human cell lines and primary cells are commonly employed for recapitulating and studying human biology in OoaC systems due to their ease of cultivation, cost-effectiveness, and biological similarities to their in vivo equivalents [100]

Immortalized cell lines, obtained through genetic alterations, can be continuously cultivated without presenting any phenotypic and genotypic variations. These cell lines proliferate quickly under relatively simple culture conditions and are valuable for optimizing various parameters of the OoaC system during the early development stage. However, they may need to fully capture human physiology, including metabolic activities, efficacy, and toxicity.

On the other hand, primary cells can be isolated from human biopsies or obtained from discarded tissues. Their use in OoaC systems to model specific organs can be advantageous, as they can generate pharmacologically reliable results regarding the toxicological response triggered by xenobiotics. The capacity of primary cells to create organ-specific microenvironments enhances the similarities in behavior between cells. While primary cells provide an improved model of human physiology compared to immortalized cell lines, their ability to simulate the complexity of an organ is limited due to factors such as reduced cell proliferation and tissue sourcing challenges [101].

### Stem Cell Engineering

To address the previously mentioned limitations, stem cells have emerged as promising targets for biomimetic models aiming to accurately predict human responses to drug treatments. Stem cells show an inherent capacity for self-renewal and controllable differentiation into specialized cell types or tissue under specific microenvironmental conditions, making them a potent source of biological tissue for OoaC systems. The most common stem cell types include embryonic stem cells (ESCs), induced pluripotent stem cells (iPSCs), and adult stem cells (ASCs). Mesenchymal stem cells (MSCs), a common type of ASCs extracted from adult tissue such as bone marrow or fat tissue, have limited application in microphysiological systems due to challenges such as their limited differentiation ability, inconsistent cell culture protocols, and unclear biological responses [102]. ESCs, derived from blastocysts or internal embryo cells, exhibit pluripotent capabilities. However, ethical considerations and regulatory restrictions surround their use [103]. iPSCs, generated through the reprogramming of adult somatic cells with specific transcription factors, offer a promising alternative. They possess the ability to differentiate into various cell types and have demonstrated uniformity over time, making them suitable for large-scale studies [104]. iPSC-derived tissue models have shown great applicability in OoaC systems, developing different iPSC differentiation protocols, and updating Good Cell Culture Practice guidelines [105]. No significant differences were observed between ESCs and iPSCs with the same genetic background regarding the levels of gene expression, surface marker expression, and morphology [106,107]. Efforts have been made to create cell line libraries comprising iPSCs from individuals without characterized diseases, different genetic backgrounds, ethnicities, and those with genetic pathologies. These cell lines hold potential for disease modeling and personalized drug screening studies, offering an alternative to costly and invasive primary tissue isolation procedures [108].

In order to enable and optimize their use in OoaC systems, enhancing the efficiency and reproducibility of the iPSC differentiation process is imperative. Achieving the desired cell types from iPSCs hinges upon exposure to specific chemical and physical stimuli. Notably, to facilitate cell maturation and mimic aging, it is essential to introduce mechanical, electrical, and other stimuli replicating the in vivo environment in which the tissue naturally resides. Consequently, some systems incorporating iPSCs still exhibit an embryonic gene expression profile, highlighting the need for further development to obtain an adult phenotype.

iPSCs have attracted significant interest in the development of tailored OoaC models due to their ability to derive patient-specific iPSCs from somatic cells. This facilitates the creation of customized systems replicating normal and diseased physiology, offering a more effective alternative to animal models. Moreover, iPSC-based systems enable personalized drug screening drugs, eliminating the need for patients to undergo invasive and challenging primary tissue isolation procedures [109,110]. Integrating iPSCs into OoaC reduces pre-clinical research time and allows for drug development based on the genetic profiles and diseases of individual patients. This approach enhances the safety and toxicity testing of new drug candidates, and emphasizes the importance of exploiting iPSCs in conjugation with OoaC systems in pharmaceutical pipeline evaluations [111,112].

The relevance of iPSCs in OoaC presents an opportunity to compare individual physiological responses to drugs among multiple patients and to develop models for studying genetic diseases, even when the responsible mutation is unknown. However, despite the considerable potential offered by these cells, significant limitations remain regarding their precise ability to replicate human organs in OoaC systems. As a result, it is crucial to implement systematic experimental methods for studying individual cell responses to external physical stimuli and changes in ECM during drug screenings and toxicity assessments. These methods should be based on patient- and disease-specific iPSCs, aiming to enhance the accuracy and reliability of OoaC models for pharmaceutical research and personalized medicine [113].

## 6. Conclusions

OoaC systems have revolutionized biomedical research by providing innovative platforms to model human physiology and disease in vitro. Over the past decade (2014–2024), significant progress has been made in successfully modeling various organs and physiological systems using OoaC devices. Using the “Pubchem” database and employing the keywords “organ-on-a-chip” AND “research articles”, around 158 research articles were published, providing valuable insights into the growing interest the scientific community has in this innovative field. The expanding research in this field is closely associated with the realization of OoaC systems’ potential across diverse disciplines, from biomedical engineering and pharmacology to regenerative medicine and drug discovery. These publications emphasize the increasing recognition of OoaC systems as powerful tools for modeling complex physiological systems and disease mechanisms in vitro, mainly replicating organs such as the liver, intestine, skin, central nervous system, or kidney. These systems have been engineered to replicate essential physiological functions, including drug metabolism, toxicity assessments, and disease emulation. Compared to traditional cell culture methods, they offer a more physiologically relevant environment, allowing for more accurate predictions of drug responses and liver pathophysiology. Additionally, multi-OoaC is already a reality, with significant progress made in successfully modeling several organs connecting, and challenges remain in replicating the full complexity of human physiology and disease in vitro.

These advances suggest the progress made in scientific exploration and highlight the substantial investment from prominent companies. This emphasizes the widespread acknowledgment of the potential of these systems. Industry leaders such as Roche, AstraZeneca, Pharmacelsus, Johnson & Johnson, and others are among the notable entities channeling resources into this domain.

Despite the advantages of this system and its significant promise in advancing biomedical research, it has drawbacks. The complexity and cost associated with developing OoaC systems are barriers to widespread adoption, as specialized expertise and a large amount of equipment are required. Moreover, current OoaC models often lack the full complexity of native tissues and organs, challenging researchers aiming to accurately replicate intricate physiological processes. Furthermore, ongoing challenges remain to scale up OoaC technology for industrial applications and ensure reproducibility across different platforms. Ethical considerations regarding the use of human cells and tissues in OoaC research and the need for regulatory approval for drug development and toxicity testing further complicate the landscape. Despite these challenges, collaborative efforts are ongoing to address these limitations and unlock the full potential of OoaC technology in revolutionizing biomedical research and drug discovery.

In conclusion, the evolution of microphysiological systems, including OoaC technology, represents a remarkable stride in biomedical research. These systems offer a unique platform for replicating human physiology, bridging the gap between traditional 2D cultures and complex in vivo models. By carefully considering parameters such as geometry, dimension, channel shape, and biological context, researchers can tailor OoaC systems to better mimic human physiology and enhance the accuracy of in vitro testing.

Furthermore, the integration of biosensors, real-time monitoring capabilities, and the ability to respond to external stimuli empower OoaCs to provide crucial insights into cellular behavior and tissue responses. This level of control and precision supports a deeper understanding of disease mechanisms and accelerates drug development and personalized medicine strategies.

Microfluidic technology provides favorable support for the development of OoaC for medical or basic science investigations. Achieving this requires the integration of diverse disciplines, spanning from molecular biology to microfabrication. Converging the knowledge obtained from biomaterials, engineering, and stem cell biology presents a significant challenge in building high-fidelity tissues with translational potential. OoaC can faithfully replicate the body’s microenvironment, incorporating critical factors such as cell–cell and cell–ECM interactions, biochemical cues, and biophysical conditions—features lacking in 2D models. Furthermore, OoaC technology aligns with ethical principles, reducing the reliance on animal models, as guided by the 3Rs principles (replacement, reduction, and refinement). Simultaneously, these 3D models are anticipated to bridge the gap between translational, pre-clinical, and clinical studies. However, successful clinical translation hinges on technological maturity, encompassing reproducibility, high-throughput analysis, compatible readout techniques, and automation to establish standardized and validated chips. A notable challenge in the industry adoption of OoaC is the manufacturing cost.

Thus, microfluidics and OoaC technology hold huge potential in drug development, single- or multi-drug analysis, biosensing, diagnosis, toxicity prediction, understanding the disease models, and pharmacodynamic and pharmacokinetic analysis.

## Figures and Tables

**Table 2:** Organ-on-a-chip: advantages and drawbacks.

**Advantages**	**Drawbacks**
✓ Research acceleration ✓ Ability to test several strategies simultaneously ✓ Close resemblance to microenvironment ✓ User-friendly ✓ Portable ✓ Integration of several MPS in a single chip ✓ Ability to integrate biosensors	✕ Surface effect ✕ Product adsorption ✕ Fluids do not mix properly

## Data Availability

The original contributions presented in the study are included in the article, further inquiries can be directed to the corresponding author.

## References

[1] Jensen C., Teng Y. (2020). Is It Time to Start Transitioning From 2D to 3D Cell Culture?. Front. Mol. Biosci..

[2] Saji Joseph J., Tebogo Malindisa S., Ntwasa M. (2019). Two-Dimensional (2D) and Three-Dimensional (3D) Cell Culturing in Drug Discovery. Cell Cult..

[3] Imamura Y., Mukohara T., Shimono Y., Funakoshi Y., Chayahara N., Toyoda M., Kiyota N., Takao S., Kono S., Nakatsura T. (2015). Comparison of 2D- and 3D-culture models as drug-testing platforms in breast cancer. Oncol. Rep..

[4] Helleberg Madsen N., Schnack Nielsen B., Larsen J., Gad M. (2022). In vitro 2D and 3D cancer models to evaluate compounds that modulate macrophage polarization. Cell. Immunol..

[5] Park Y., Huh K.M., Kang S.W. (2021). Applications of biomaterials in 3d cell culture and contributions of 3d cell culture to drug development and basic biomedical research. Int. J. Mol. Sci..

[6] Chin L.K., Lee C.-H., Chen B.-C. (2016). Imaging live cells at high spatiotemporal resolution for lab-on-a-chip applications. Lab Chip.

[7] Leung C.M., de Haan P., Ronaldson-Bouchard K., Kim G.A., Ko J., Rho H.S., Chen Z., Habibovic P., Jeon N.L., Takayama S. (2022). A guide to the organ-on-a-chip. Nat. Rev. Methods Prim..

[8] Stock K., Estrada M.F., Vidic S., Gjerde K., Rudisch A., Santo V.E., Barbier M., Blom S., Arundkar S.C., Selvam I. (2016). Capturing tumor complexity in vitro: Comparative analysis of 2D and 3D tumor models for drug discovery. Sci. Rep..

[9] Bhatia S.N., Ingber D.E. (2014). Microfluidic organs-on-chips. Nat. Biotechnol..

[10] Ware B.R., Khetani S.R. (2017). Engineered Liver Platforms for Different Phases of Drug Development. Trends Biotechnol..

[11] Zhang B., Radisic M. (2017). Organ-on-A-chip devices advance to market. Lab Chip.

[12] Messner S., Agarkova I., Moritz W., Kelm J.M. (2013). Multi-cell type human liver microtissues for hepatotoxicity testing. Arch. Toxicol..

[13] Liu C., Oikonomopoulos A., Sayed N., Wu J.C. (2018). Modeling human diseases with induced pluripotent stem cells: From 2D to 3D and beyond. Development.

[14] Huh D., Hamilton G.A., Ingber D.E. (2011). From 3D cell culture to organs-on-chips. Trends Cell Biol..

[15] Singh Dolt K., Hammachi F., Kunath T. (2017). Modeling Parkinson’s disease with induced pluripotent stem cells harboring α-synuclein mutations. Brain Pathol..

[16] Wallet M.A., Santostefano K.E., Terada N., Brusko T.M. (2017). Isogenic cellular systems model the impact of genetic risk variants in the pathogenesis of type 1 diabetes. Front. Endocrinol..

[17] Franco S.S., Szczesna K., Iliou M.S., Al-Qahtani M., Mobasheri A., Kobolák J., Dinnyés A. (2016). In vitro models of cancer stem cells and clinical applications. BMC Cancer.

[18] Jiang Y., Park P., Hong S.M., Ban K. (2018). Maturation of cardiomyocytes derived from human pluripotent stem cells: Current strategies and limitations. Mol. Cells.

[19] Costa L., Reis R.L., Silva-Correia J., Oliveira J.M. (2020). Microfluidics for Angiogenesis Research. Biomaterials-and Microfluidics-Based Tissue Engineered 3D Models.

[20] Edmondson R., Broglie J.J., Adcock A.F., Yang L. (2014). Three-Dimensional Cell Culture Systems and Their Applications in Drug Discovery and Cell-Based Biosensors. Assay Drug Dev. Techno..

[21] Skardal A., Devarasetty M., Rodman C., Atala A., Soker S. (2015). Liver-tumor hybrid organoids for modeling tumor growth and drug response in vitro. Ann. Biomed. Eng..

[22] Devarasetty M., Skardal A., Cowdrick K., Marini F., Soker S. (2017). Bioengineered submucosal organoids for in vitro modeling of colorectal cancer. Tissue Eng. Part A.

[23] Kim M., Mun H., Sung C.O., Cho E.J., Jeon H.-J., Chun S.-M., Jung D.J., Shin T.H., Jeong G.S., Kim D.K. (2019). Patient-derived lung cancer organoids as in vitro cancer models for therapeutic screening. Nat. Commun..

[24] Kunisaki S.M., Jiang G., Biancotti J.C., Ho K.K.Y., Dye B.R., Liu A.P., Spence J.R. (2021). Human induced pluripotent stem cell-derived lung organoids in an ex vivo model of the congenital diaphragmatic hernia fetal lung. Stem Cells Transl. Med..

[25] Kim H.K., Kim H., Lee M.K., Choi W.H., Jang Y., Shin J.S., Park J.-Y., Bae D.H., Hyun S.-I., Kim K.H. (2022). Generation of human tonsil epithelial organoids as an ex vivo model for SARS-CoV-2 infection. Biomaterials.

[26] Camp J.G., Badsha F., Florio M., Kanton S., Gerber T., Wilsch-Bräuninger M., Lewitus E., Sykes A., Hevers W., Lancaster M. (2015). Human cerebral organoids recapitulate gene expression programs of fetal neocortex development. Proc. Natl. Acad. Sci. USA.

[27] Ham O., Jin Y.B., Kim J., Lee M.-O. (2020). Blood vessel formation in cerebral organoids formed from human embryonic stem cells. Biochem. Biophys. Res. Commun..

[28] Berkers G., van Mourik P., Vonk A.M., Kruisselbrink E., Dekkers J.F., de Winter-de Groot K.M., Arets H.G.M., Marck-van der Wilt R.E.P., Dijkema J.S., Vanderschuren M.M. (2019). Rectal organoids enable personalized treatment of cystic fibrosis. Cell Rep..

[29] Kim J., Koo B.-K., Knoblich J.A. (2020). Human organoids: Model systems for human biology and medicine. Nat. Rev. Mol. Cell Biol..

[30] Tanataweethum N., Zelaya A., Yang F., Cohen R.N., Brey E.M., Bhushan A. (2018). Establishment and characterization of a primary murine adipose tissue-chip. Biotechnol. Bioeng..

[31] Qian F., Huang C., Lin Y.-D., Ivanovskaya A.N., O’Hara T.J., Booth R.H., Creek C.J., Enright H.A., Soscia D.A., Belle A.M. (2017). Simultaneous electrical recording of cardiac electrophysiology and contraction on chip. Lab Chip.

[32] van der Helm M.W., Odijk M., Frimat J.-P., van der Meer A.D., Eijkel J.C.T., van den Berg A., Segerink L.I. (2016). Direct quantification of transendothelial electrical resistance in organs-on-chips. Biosens. Bioelectron..

[33] Moutaux E., Charlot B., Genoux A., Saudou F., Cazorla M. (2018). An integrated microfluidic/microelectrode array for the study of activity-dependent intracellular dynamics in neuronal networks. Lab Chip.

[34] Liu G., Qi M., Hutchinson M.R., Yang G., Goldys E.M. (2016). Recent advances in cytokine detection by immunosensing. Biosens. Bioelectron..

[35] Weltin A., Hammer S., Noor F., Kaminski Y., Kieninger J., Urban G.A. (2017). Accessing 3D microtissue metabolism: Lactate and oxygen monitoring in hepatocyte spheroids. Biosens. Bioelectron..

[36] Moya A., Ortega-Ribera M., Guimerà X., Sowade E., Zea M., Illa X., Ramon E., Villa R., Gracia-Sancho J., Gabriel G. (2018). Online oxygen monitoring using integrated inkjet-printed sensors in a liver-on-a-chip system. Lab Chip.

[37] Liu H., MacQueen L.A., Usprech J.F., Maleki H., Sider K.L., Doyle M.G., Sun Y., Simmons C.A. (2018). Microdevice arrays with strain sensors for 3D mechanical stimulation and monitoring of engineered tissues. Biomaterials.

[38] van de Wijdeven R., Ramstad O.H., Valderhaug V.D., Köllensperger P., Sandvig A., Sandvig I., Halaas Ø. (2019). A novel lab-on-chip platform enabling axotomy and neuromodulation in a multi-nodal network. Biosens. Bioelectron..

[39] Doherty E.L., Aw W.Y., Hickey A.J., Polacheck W.J. (2021). Microfluidic and Organ-on-a-Chip Approaches to Investigate Cellular and Microenvironmental Contributions to Cardiovascular Function and Pathology. Front. Bioeng. Biotechnol..

[40] Wikswo J.P. (2014). The relevance and potential roles of microphysiological systems in biology and medicine. Exp. Biol. Med..

[41] El-Ali J., Sorger P.K., Jensen K.F. (2006). Cells on chips. Nature.

[42] Balijepalli A., Sivaramakrishan V. (2017). Organs-on-chips: Research and commercial perspectives. Drug Discov. Today.

[43] Xu Z., Gao Y., Hao Y., Li E., Wang Y., Zhang J., Wang W., Gao Z., Wang Q. (2013). Application of a microfluidic chip-based 3D co-culture to test drug sensitivity for individualized treatment of lung cancer. Biomaterials.

[44] Sosa-Hernández J.E., Villalba-Rodríguez A.M., Romero-Castillo K.D., Aguilar-Aguila-Isaías M.A., García-Reyes I.E., Hernández-Antonio A., Ahmed I., Sharma A., Parra-Saldívar R., Iqbal H.M.N. (2018). Organs-on-a-chip module: A review from the development and applications perspective. Micromachines.

[45] Henry O.Y.F., Villenave R., Cronce M.J., Leineweber W.D., Benz M.A., Ingber D.E. (2017). Organs-on-chips with integrated electrodes for trans-epithelial electrical resistance (TEER) measurements of human epithelial barrier function. Lab Chip.

[46] Koutsouras D.A., Perrier R., Villarroel Marquez A., Pirog A., Pedraza E., Cloutet E., Renaud S., Raoux M., Malliaras G.G., Lang J. (2017). Simultaneous monitoring of single cell and of micro-organ activity by PEDOT:PSS covered multi-electrode arrays. Mater. Sci. Eng. C.

[47] Maoz B.M., Herland A., Henry O.Y.F., Leineweber W.D., Yadid M., Doyle J., Mannix R., Kujala V.J., Fitzgerald E.A., Parker K.K. (2017). Organs-on-Chips with combined multi-electrode array and transepithelial electrical resistance measurement capabilities. Lab Chip.

[48] Riahi R., Shaegh S.A.M., Ghaderi M., Zhang Y.S., Shin S.R., Aleman J., Massa S., Kim D., Dokmeci M.R., Khademhosseini A. (2016). Automated microfluidic platform of bead-based electrochemical immunosensor integrated with bioreactor for continual monitoring of cell secreted biomarkers. Sci. Rep..

[49] Lou Y.R., Leung A.W. (2018). Next generation organoids for biomedical research and applications. Biotechnol. Adv..

[50] Dutta D., Heo I., Clevers H. (2017). Disease modeling in stem cell-derived 3D organoid systems. Trends Mol. Med..

[51] Andrews M.G., Kriegstein A.R. (2022). Challenges of organoid research. Annu. Rev. Neurosci..

[52] Takebe T., Zhang B., Radisic M. (2017). Synergistic Engineering: Organoids Meet Organs-on-a-Chip. Cell Stem Cell.

[53] Takebe T., Enomura M., Yoshizawa E., Kimura M., Koike H., Ueno Y., Matsuzaki T., Yamazaki T., Toyohara T., Osafune K. (2015). Vascularized and complex organ buds from diverse tissues via mesenchymal cell-driven condensation. Cell Stem Cell.

[54] Zhang Y.S., Arneri A., Bersini S., Shin S.R., Zhu K., Goli-Malekabadi Z., Aleman J., Colosi C., Busignani F., Dell’Erba V. (2016). Bioprinting 3D microfibrous scaffolds for engineering endothelialized myocardium and heart-on-a-chip. Biomaterials.

[55] Xu H., Li Z., Yu Y., Sizdahkhani S., Ho W.S., Yin F., Wang L., Zhu G., Zhang M., Jiang L. (2016). A dynamic in vivo-like organotypic blood-brain barrier model to probe metastatic brain tumors. Sci. Rep..

[56] LiáJeon N. (2015). Microfluidic vascularized bone tissue model with hydroxyapatite-incorporated extracellular matrix. Lab Chip.

[57] George M. (2006). Whitesides The origins and the future of microfluidics. Nature.

[58] Barata D., van Blitterswijk C., Habibovic P. (2016). High-throughput screening approaches and combinatorial development of biomaterials using microfluidics. Acta Biomater..

[59] Du G., Fang Q., den Toonder J.M.J. (2016). Microfluidics for cell-based high throughput screening platforms—A review. Anal. Chim. Acta.

[60] Torino S., Corrado B., Iodice M., Coppola G. (2018). PDMS-based microfluidic devices for cell culture. Inventions.

[61] McDonald J.C., Whitesides G.M. (2002). Poly(dimethylsiloxane) as a material for fabricating microfluidic devices. Acc. Chem. Res..

[62] Ahmed I., Iqbal H., Akram Z. (2018). Microfluidics engineering: Recent trends, valorization, and applications. Arab. J. Sci. Eng..

[63] Prabhakar P., Sen R.K., Dwivedi N., Khan R., Solanki P.R., Srivastava A.K., Dhand C. (2021). 3D-Printed microfluidics and potential biomedical applications. Front. Nanotechnol..

[64] Waheed S., Cabot J.M., Macdonald N.P., Lewis T., Guijt R.M., Paull B., Breadmore M.C. (2016). 3D printed microfluidic devices: Enablers and barriers. Lab Chip.

[65] Yi H.G., Lee H., Cho D.W. (2017). 3D printing of organs-on-chips. Bioengineering.

[66] Bhattacharjee N., Urrios A., Kang S., Folch A. (2016). The upcoming 3D-printing revolution in microfluidics. Lab Chip.

[67] Kundu A., McCoy L., Azim N., Nguyen H., Didier C.M., Ausaf T., Sharma A.D., Curley J.L., Moore M.J., Rajaraman S. (2020). Fabrication and characterization of 3D Printed, 3D microelectrode arrays for interfacing with a peripheral nerve-on-a-chip. ACS Biomater. Sci. Eng..

[68] Pradhan S., Hassani I., Clary J.M., Lipke E.A. (2016). Polymeric biomaterials for in vitro cancer tissue engineering and drug testing applications. Tissue Eng.-Part B Rev..

[69] Miranda I., Souza A., Sousa P., Ribeiro J., Castanheira E.M.S., Lima R., Minas G. (2022). Properties and applications of PDMS for biomedical engineering: A review. J. Funct. Biomater..

[70] Wen H., Yu Y., Zhu G., Jiang L., Qin J. (2015). A droplet microchip with substance exchange capability for the developmental study of C. elegans. Lab Chip.

[71] Campbell S.B., Wu Q., Yazbeck J., Liu C., Okhovatian S., Radisic M. (2020). Beyond polydimethylsiloxane: Alternative materials for fabrication of organ-on-a-chip devices and microphysiological systems. ACS Biomater. Sci. Eng..

[72] Berthier E., Young E.W.K., Beebe D. (2012). Engineers are from PDMS-land, Biologists are from Polystyrenia. Lab Chip.

[73] Choi J., Kim S., Jung J., Lim Y., Kang K., Park S., Kang S. (2011). Wnt5a-mediating neurogenesis of human adipose tissue-derived stem cells in a 3D microfluidic cell culture system. Biomaterials.

[74] Shi Y., Ma J., Zhang X., Li H., Jiang L., Qin J. (2015). Hypoxia combined with spheroid culture improves cartilage specific function in chondrocytes. Integr. Biol..

[75] Toh Y.C., Lim T.C., Tai D., Xiao G., Van Noort D., Yu H. (2009). A microfluidic 3D hepatocyte chip for drug toxicity testing. Lab Chip.

[76] Chung B.G., Lee K.-H., Khademhosseini A., Lee S.-H. (2012). Microfluidic fabrication of microengineered hydrogels and their application in tissue engineering. Lab Chip.

[77] Walker G.M., Zeringue H.C., Beebe D.J. (2004). Microenvironment design considerations for cellular scale studies. Lab Chip.

[78] Yamada M., Hori A., Sugaya S., Yajima Y., Utoh R., Yamato M., Seki M. (2015). Cell-sized condensed collagen microparticles for preparing microengineered composite spheroids of primary hepatocytes. Lab Chip.

[79] Sackmann E.K., Fulton A.L., Beebe D.J. (2014). The present and future role of microfluidics in biomedical research. Nature.

[80] Shen B. (2015). A New Golden Age of Natural Products Drug Discovery. Cell.

[81] Brown J.A., Pensabene V., Markov D.A., Allwardt V., Neely M.D., Shi M., Britt C.M., Hoilett O.S., Yang Q., Brewer B.M. (2015). Recreating blood-brain barrier physiology and structure on chip: A novel neurovascular microfluidic bioreactor. Biomicrofluidics.

[82] Wang Y., Ma J., Li N., Wang L., Shen L., Sun Y., Wang Y., Zhao J., Wei W., Ren Y. (2017). Microfluidic engineering of neural stem cell niches for fate determination. Biomicrofluidics.

[83] Imparato G., Urciuolo F., Netti P.A. (2022). Organ on Chip Technology to Model Cancer Growth and Metastasis. Bioengineering.

[84] Caballero D., Kaushik S., Correlo V.M., Oliveira J.M., Reis R.L., Kundu S.C. (2017). Organ-on-chip models of cancer metastasis for future personalized medicine: From chip to the patient. Biomaterials.

[85] Liu W., Song J., Du X., Zhou Y., Li Y., Li R., Lyu L., He Y., Hao J., Ben J. (2019). AKR1B10 (Aldo-keto reductase family 1 B10) promotes brain metastasis of lung cancer cells in a multi-organ microfluidic chip model. Acta Biomater..

[86] Zuchowska A., Skorupska S. (2022). Multi-organ-on-chip approach in cancer research. Organs-A-Chip.

[87] Joanne Wang C., Li X., Lin B., Shim S., Ming G.L., Levchenko A. (2008). A microfluidics-based turning assay reveals complex growth cone responses to integrated gradients of substrate-bound ECM molecules and diffusible guidance cues. Lab Chip.

[88] Baker B.M., Trappmann B., Stapleton S.C., Toro E., Chen C.S. (2013). Microfluidics embedded within extracellular matrix to define vascular architectures and pattern diffusive gradients. Lab Chip.

[89] Shakeri A., Sun N., Badv M., Didar T.F. (2017). Generating 2-dimensional concentration gradients of biomolecules using a simple microfluidic design. Biomicrofluidics.

[90] Kamei K., Mashimo Y., Koyama Y., Fockenberg C., Nakashima M., Nakajima M., Li J., Chen Y. (2015). 3D printing of soft lithography mold for rapid production of polydimethylsiloxane-based microfluidic devices for cell stimulation with concentration gradients. Biomed. Microdevices.

[91] Shin Y., Jeon J.S., Han S., Jung G.S., Shin S., Lee S.H., Sudo R., Kamm R.D., Chung S. (2011). In vitro 3D collective sprouting angiogenesis under orchestrated ANG-1 and VEGF gradients. Lab Chip.

[92] Grant J., Lee E., Almeida M., Kim S., LoGrande N., Goyal G., Sesay A.M., Breault D.T., Prantil-Baun R., Ingber D.E. (2022). Establishment of physiologically relevant oxygen gradients in microfluidic organ chips. Lab Chip.

[93] Ribas J., Zhang Y.S., Pitrez P.R., Leijten J., Miscuglio M., Rouwkema J., Dokmeci M.R., Nissan X., Ferreira L., Khademhosseini A. (2017). Biomechanical strain exacerbates inflammation on a progeria-on-a-chip model. Small.

[94] Song J.W., Munn L.L. (2011). Fluid forces control endothelial sprouting. Proc. Natl. Acad. Sci. USA.

[95] Guenat O.T., Berthiaume F. (2018). Incorporating mechanical strain in organs-on-a-chip: Lung and skin. Biomicrofluidics.

[96] Thompson C.L., Fu S., Heywood H.K., Knight M.M., Thorpe S.D. (2020). Mechanical stimulation: A crucial element of organ-on-chip models. Front. Bioeng. Biotechnol..

[97] Feric N.T., Pallotta I., Singh R., Bogdanowicz D.R., Gustilo M.M., Chaudhary K.W., Willette R.N., Chendrimada T.P., Xu X., Graziano M.P. (2019). Engineered cardiac tissues generated in the biowire II: A platform for human-based drug discovery. Toxicol. Sci..

[98] Lee D., Erickson A., You T., Dudley A.T., Ryu S. (2018). Pneumatic microfluidic cell compression device for high-throughput study of chondrocyte mechanobiology. Lab Chip.

[99] Occhetta P., Mainardi A., Votta E., Vallmajo-Martin Q., Ehrbar M., Martin I., Barbero A., Rasponi M. (2019). Hyperphysiological compression of articular cartilage induces an osteoarthritic phenotype in a cartilage-on-a-chip model. Nat. Biomed. Eng..

[100] Workman M.J., Gleeson J.P., Troisi E.J., Estrada H.Q., Kerns S.J., Hinojosa C.D., Hamilton G.A., Targan S.R., Svendsen C.N., Barrett R.J. (2018). Enhanced Utilization of Induced Pluripotent Stem Cell–Derived Human Intestinal Organoids Using Microengineered Chips. Cmgh.

[101] Luni C., Serena E., Elvassore N. (2014). Human-on-chip for therapy development and fundamental science. Curr. Opin. Biotechnol..

[102] Wagner W., Ho A.D. (2007). Mesenchymal stem cell preparations-comparing apples and oranges. Stem Cell Rev..

[103] Mushahary D., Spittler A., Kasper C., Weber V., Charwat V. (2018). Isolation, cultivation, and characterization of human mesenchymal stem cells. Cytom. Part A.

[104] Sayed N., Liu C., Wu J.C. (2016). Translation of Human-Induced Pluripotent Stem Cells from Clinical Trial in a Dish to Precision Medicine. J. Am. Coll. Cardiol..

[105] Geraili A., Jafari P., Hassani M.S., Araghi B.H., Mohammadi M.H., Ghafari A.M., Tamrin S.H., Modarres H.P., Kolahchi A.R., Ahadian S. (2018). Controlling differentiation of stem cells for developing personalized organ-on-chip platforms. Adv. Healthc. Mater..

[106] Narsinh K.H., Plews J., Wu J.C. (2011). Comparison of human induced pluripotent and embryonic stem cells: Fraternal or identical twins?. Mol. Ther..

[107] Wagner W., Feldmann R.E., Seckinger A., Maurer M.H., Wein F., Blake J., Krause U., Kalenka A., Bürgers H.F., Saffrich R. (2006). The heterogeneity of human mesenchymal stem cell preparations-Evidence from simultaneous analysis of proteomes and transcriptomes. Exp. Hematol..

[108] Pamies D., Bal-Price A., Chesné C., Coecke S., Dinnyes A., Eskes C., Grillari R., Gstraunthaler G., Hartung T., Jennings P. (2018). Advanced Good Cell Culture Practice for human primary, stem cell-derived and organoid models as well as microphysiological systems. ALTEX.

[109] Azizipour N., Avazpour R., Rosenzweig D.H., Sawan M., Ajji A. (2020). Evolution of biochip technology: A review from lab-on-a-chip to organ-on-a-chip. Micromachines.

[110] Skardal A., Shupe T., Atala A. (2016). Organoid-on-a-chip and body-on-a-chip systems for drug screening and disease modeling. Drug Discov. Today.

[111] Scott C.W., Peters M.F., Dragan Y.P. (2013). Human induced pluripotent stem cells and their use in drug discovery for toxicity testing. Toxicol. Lett..

[112] Esch E.W., Bahinski A., Huh D. (2015). Organs-on-chips at the frontiers of drug discovery. Nat. Rev. Drug Discov..

[113] Esch M.B., Prot J.-M., Wang Y.I., Miller P., Llamas-Vidales J.R., Naughton B.A., Applegate D.R., Shuler M.L. (2015). Multi-cellular 3D human primary liver cell culture elevates metabolic activity under fluidic flow. Lab Chip.

